# Progestogen-only contraception use during breastfeeding: an updated systematic review

**DOI:** 10.1136/bmjsrh-2025-202837

**Published:** 2025-11-03

**Authors:** Angeline Ti, Sylvia Ayieko, Mercedes Bonet

**Affiliations:** 1 Wellstar Douglas Family Medicine Residency Program, Douglasville, Georgia, USA; 2 The University of Iowa College of Public Health, Iowa City, Iowa, USA; 3 UNDP-UNFPA-UNICEF-WHO-World Bank Special Programme of Research, Development and Research Training in Human Reproduction (HRP), Department of Sexual and Reproductive Health and Research, World Health Organization, Geneva, Switzerland

**Keywords:** hormonal contraception, family planning services

## Abstract

**Objectives:**

This systematic review aimed to update a 2016 review and answer two questions: (1) Among breastfeeding women, does the use of progestogen-only contraception (POC) (ie, pills, injectables, implants, hormonal intrauterine devices) increase the risk of poor breastfeeding or infant outcomes compared with those not using POC? (2) Among breastfeeding women, does the initiation of POC before 6 weeks postpartum increase the risk of poor breastfeeding or infant outcomes compared with the initiation of POC at 6 weeks or later?

**Methods:**

We searched multiple databases (MEDLINE, EMBASE, Cochrane, ClinicalTrials.gov and CINAHL) from inception to 6 September 2023. We extracted data and assessed risk of bias (RoB) for each study and certainty of evidence for each outcome.

**Results:**

Sixty-one articles met the inclusion criteria; 11 were newly identified since the previous review, most with high RoB. Nine new randomised and non-randomised studies assessing breastfeeding and/or infant outcomes met the inclusion criteria for Question 1. Two new randomised studies assessing breastfeeding and/or infant outcomes met the inclusion criteria for Question 2, examining early versus late initiation of the implant. One new article for each objective included preterm infants. For both questions, studies continue to find no significant adverse effects on breastfeeding (eg, continuation, supplementation, duration) or infant (eg, growth, illness) outcomes. The certainty of evidence ranged from very low to moderate across outcomes.

**Conclusions:**

This updated systematic review provides additional evidence for the safety of POC use during breastfeeding. Newly identified studies are consistent with the prior review in suggesting no consistent findings of adverse effects, while adding evidence for preterm infants.

**WHAT IS ALREADY KNOWN ON THIS TOPIC**

- A systematic review published in 2016 examining breastfeeding and the use of progestogen-only contraception (POC) generally found no evidence of adverse breastfeeding outcomes or negative infant health outcomes.

**WHAT THIS STUDY ADDS**

- This updated systematic review identified additional evidence that continues to support the safety of POC during breastfeeding. Among 11 newly identified studies, POC use was not associated with significant adverse effects on breastfeeding or infant outcomes.

**HOW THIS STUDY MIGHT AFFECT RESEARCH, PRACTICE OR POLICY**

- Clinicians and policymakers can use this evidence on the safety of POC during breastfeeding when counselling patients and considering policies and programmes. Future research can further assess the safety of POC use among populations with risk factors for breastfeeding difficulties.

## Introduction

 Contraception is an important component of healthcare that helps facilitate reproductive rights and autonomy and is crucial during the postpartum period.^1 2^ The WHO and other organisations recommend exclusive breastfeeding for the first 6 months postpartum and continued breastfeeding up to 2 years or more.^1 3^ Breastfeeding (either directly feeding at the breast or feeding expressed milk) has significant health benefits for the individual breastfeeding and the infant.^3^ For those who are directly breastfeeding for the majority of feeds within the first 6 months postpartum and amenorrheic, the lactational amenorrhea method (LAM) is 98% effective as contraception;^4 5^ however, many who breastfeed may not meet LAM criteria or may not wish to rely on LAM.^6^ Therefore, many who breastfeed may also use additional forms of contraception, including progestogen-only contraception (POC).^7^

The rapid decrease of progesterone after delivery plays a significant role in lactogenesis.^8^ There is theoretical concern that exogenous progestogens could interfere with breastfeeding, especially for those at increased risk of delayed lactogenesis.^9^ For the infant, exogenous progestogens can be transferred from breast milk and detected in infant serum,^10^ while progesterone does not hold similar risks of infant transfer.^11^ Rodent studies have found progesterone receptors are common in the developing brain,^12^ and there may be concerns that exogenous progestogens could affect infant development.^13^

A prior systematic review published in 2016 identified 50 articles and generally failed to show adverse breastfeeding or infant health effects associated with the use of POC.^14^ The 2016 review and this current review were conducted to inform the WHO Medical Eligibility Criteria for Contraceptive Use (MEC), which provides recommendations on the safety of POC initiated before 6 weeks postpartum or at 6 weeks or later.^15^ We aimed to update the previous review and answer two research questions: (1) Among women who breastfeed, does the use of POC, including progestogen-only pills (POPs), injectables, implants and hormonal intrauterine devices (IUDs), increase the risk of poor breastfeeding or infant outcomes compared with those who do not use a POC? (2) Among women who breastfeed, does the initiation of POC before 6 weeks postpartum increase the risk of poor breastfeeding or infant outcomes compared with the initiation of POC at 6 weeks postpartum or later?

## Methods

We followed the Preferred Reporting Items for Systematic Reviews and Meta-Analyses (PRISMA) guidelines for reporting this review.^16^ Details of the protocol for this systematic review were registered on PROSPERO (Registration ID: CRD42023471081).^17^

### Eligibility Criteria

We included all comparative study designs, including primary data reported in randomised trials, non-randomised trials, comparative cohort studies and case–control studies published in any language and setting. We excluded unpublished data, conference abstracts, dissertations, case reports or case series.

We included primary articles reporting on studies of breastfeeding participants taking an exogenous progestogen (oral, injectable, implantable or hormonal IUDs) for contraception. The 2016 review also included progesterone pellets; however, we excluded these as they are no longer available. For Question 1, we included studies where POC was initiated at any time postpartum compared with any non-POC contraception or no contraception. For Question 2, we included studies where POC was initiated before 6 weeks postpartum compared with initiation at 6 weeks or later. MEC recommendations are divided by timing postpartum (<6 weeks or ≥6 weeks), related to when lactation is fully established and to the timing of a routine postpartum visit. We included articles that reported on breastfeeding outcomes (duration, discontinuation, objective change in milk supply or use of supplementation with formula), and infant growth or development (comparative objective measures), or infant illness (provider diagnosis or chart review).

### Search Strategy

We worked with a research librarian to develop a comprehensive search strategy (online supplemental file 1). The search was conducted in MEDLINE, EMBASE, Cochrane, ClinicalTrials.gov and CINAHL from database inception to 6 September 2023. The previous review only searched PubMed.

### Study Selection and Data Abstraction

**Figure 1:**
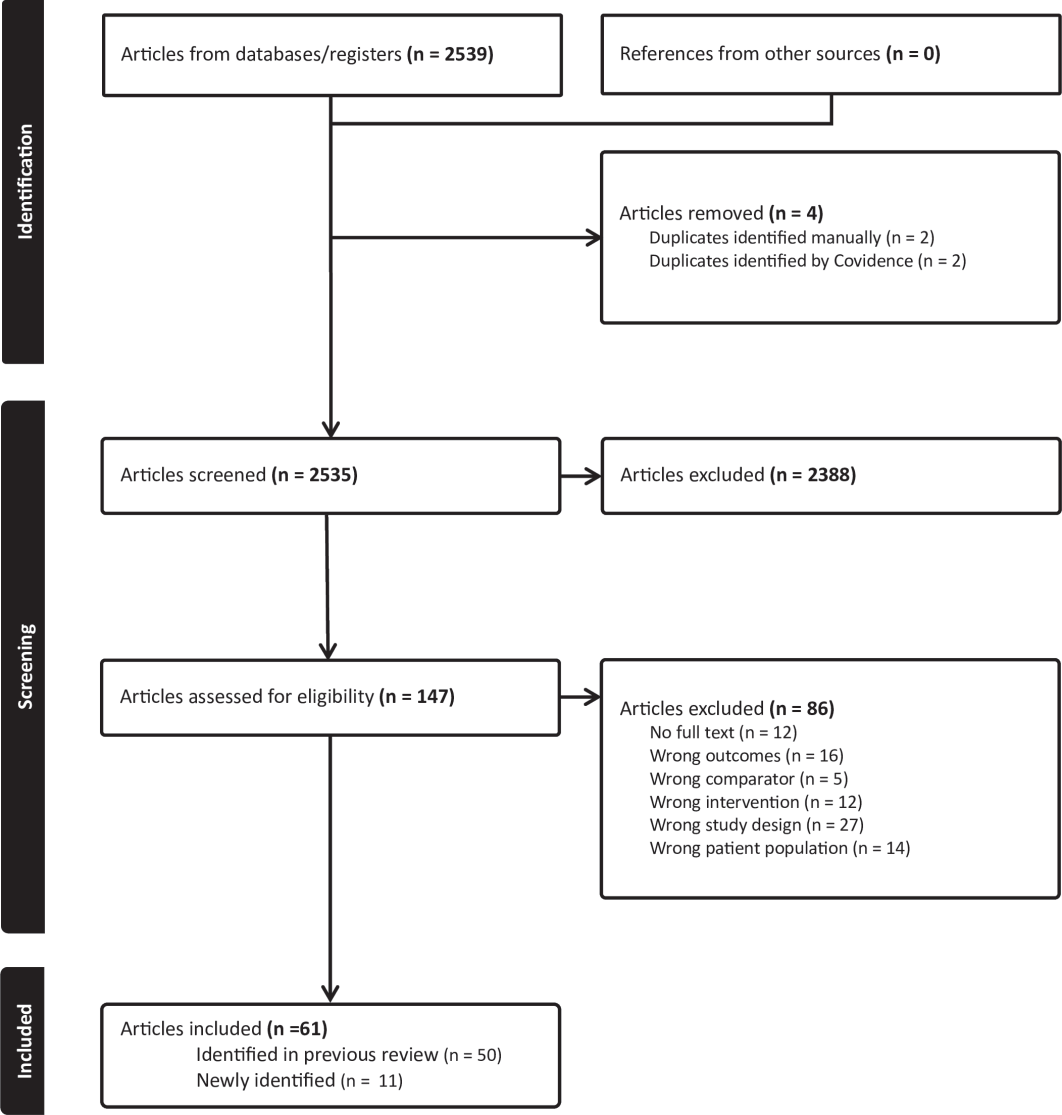
Preferred Reporting Items for Systematic Reviews and Meta-Analyses (PRISMA) flow diagram of the search process for the systematic review on the use of progestogen-only contraception during breastfeeding.

Using Covidence,^17^ each title and abstract identified in the search was reviewed independently by two researchers to determine which required full-text review. The full text of the relevant articles was then evaluated independently by two researchers. Discrepancies in article selection were resolved by discussion with senior researchers. Data from newly identified studies were extracted using standardised data tables by the lead and confirmed by another researcher. We developed a PRISMA flow diagram documenting the search (figure 1).

### Study Synthesis and Assessment

Data from the newly identified articles were described narratively along with a summary of the evidence from the 2016 review. Meta-analysis was not possible due to heterogeneity of populations, exposures and outcomes. For each new article, two researchers independently used the Cochrane RoB assessment tool (RoB2) to assess risk of bias (RoB) in randomised clinical trials (RCTs)^18^ and a modified version to assess non-randomised studies.

To assess the certainty of the evidence (CoE), we used the Grading of Recommendations Assessment, Development and Evaluation (GRADE) approach^19^ with the guidance of a GRADE methodologist. The CoE for individual outcomes was assessed including the newly and previously identified articles and was rated as high, moderate, low or very low. The rating of evidence from RCTs started at high certainty, and non-randomised trials and observational studies started at low, and then were adjusted according to assessments of RoB, indirectness, imprecision and inconsistency.

## Results

The search identified 2535 unique articles, 2388 of which were excluded based on review of title and abstract and 86 of which were excluded based on full-text review; 61 articles (59 studies) met the inclusion criteria (figure 1, online supplemental file 2). Of these, 50 were included in the prior review^14^ and 11 were newly identified,^20^,^30^ though 7 were published during the search period of the previous review.^21^,^2426^ For each research question, newly identified articles are described in detail, presented by specific contraceptive method for breastfeeding then infant outcomes. A summary of the overall body of evidence is provided for each research question.

### Question 1: Among women who breastfeed, does the use of POC increase the risk of poor breastfeeding or infant outcomes compared with those who do not use POC?

Nine newly identified articles addressed Question 1 and assessed breastfeeding and/or infant outcomes for POC users versus non-users who were breastfeeding (table 1).^20^,^28^ Five reported on POPs,^21 23 24 27 28^ two reported on injectables^25 26^ and two reported on implants.^20 22^ One included very-low birth weight (VLBW) infants^25^ and the remainder included healthy, term infants. Two studies were conducted in high-income countries,^22 25^ and the remainder were from low- or middle-income countries.

**Table 1:** Characteristics of newly identified studies in a systematic review update on progestogen-only contraception (POC) use during breastfeeding addressing the research question ‘Among women who breastfeed, does the use of POC increase the risk of poor breastfeeding or infant outcomes compared with those who do not use POC?’

Author, year, funding	Study design, location, population	Interventions	Outcomes, follow-up duration	Results	Risk of bias
Braga, 2015^20^ São Paulo Research Foundation, National Council for Scientific and Technological Development	RCT Brazil PP adult healthy women after normal delivery and their infants n=24	Randomised within 48 hours PP:Implant (etonogestrel)=12 Control (no contraceptive method)=12	Breastfeeding outcomes BF continuation through 6 weeks PP Amount of BM ingested by infant (measured using deuterium oxide [D20] ingested by mother) at 29 and 42 days Infant outcomes Infant weight at 6 weeks	Breastfeeding results Through 6 weeks PP: implant, n=11 (92%); control, n=8 (80%), no p-value At 29 days, implant: mean=343.6±102.5 mL/day; control: mean=388.2 ±170.4 mL/day, p=0.54 At 42 days, implant: mean=775±277.6 mL/day; control: mean=815.4±184.1 mL/day, p=0.63 Infant results Implant=4817.1 g (SD=534.9); control=4808 g (SD=653.7), p=0.88	Moderate
Delgado Betancourt, 1984^21^ [Bibr R21] None specified	Non-randomised clinical trial Mexico PP healthy women aged 20–35 years, who delivered a healthy singleton n=231	Selected method within 1 week PP: POP (lynestrenol)=75 Multiload Cu-IUD=76 Non-hormonal non-IUD control=80	Breastfeeding outcomes Supplementation Follow-up: 6 months Infant outcomes Infant weight at 1, 2, 3, 4, 5 and 6 months Infant length at 1, 2, 3, 4, 5 and 6 months	Breastfeeding results No significant differences in number of supplementary feeds (data not shown) One in each group dropped out due to inadequate lactation Infant results Mean weight (g): POP (4194, 4987, 5761, 6352, 7365, 8177); IUD (4086, 4794, 5679, 6452, 7166, 7996); control (4069, 4818, 5565, 6200, 6881, 7604) Mean length (cm): POP (54.6, 57.2, 59.5, 62.1, 65.0, 67.9); IUD (54.1, 57.2, 59.7, 62.2, 64.8, 67.8); control (53.6, 56.4, 58.8, 61.4, 63.9, 66.9)	High
Diaz,1985^22^ Ford Foundation, Population Council	Non-randomised clinical trial Chile Healthy women aged 18–35 years, with a normal, term pregnancy and a healthy newborn n=200	Selected method on PP day 55±3: implant (six-rod LNG)=100 Cu-IUD (TCu380Ag)=100	Breastfeeding outcomes Exclusivity (defined as BM only to 6 months and no dairy supplements after 6 months) at 12 months Supplementation (for infant growth concerns or maternal decision) through 12 months Infant outcomes Infant weight at delivery, 31, 56, 92, 122, 153, 183, 243, 305 and 366 days (±2–4 days) among breastfed infants	Breastfeeding results Implant group with lower proportions of exclusive BF compared with Cu-IUD (no numerical data provided), (p<0.02) at month 12 Implant group with higher frequency of supplementation due to poor infant growth (no data provided) Infant results Average weight, g (SD): at delivery, implant=3243 (243), IUD=3318 (298); 31 days, implant=4272 (383), IUD=4378 (407); 56 days, implant=5171 (492), IUD=5273 (511); 92 days, implant=6135 (613), IUD=6291 (654); 122 days, implant=6847 (687), IUD=7096 (760), p<0.05; 153 days, implant=7429 (705), IUD=7815 (812), p<0.05; 183 days, implant=7978 (683), IUD=8345 (876), p<0.05; 243 days, implant=8991 (772), IUD=9254 (946); 305 days, implant=9722 (864), IUD=10 111 (950); 366 days, implant=10 724 (678), IUD=10 819 (873)	High
Dutta, 2013^23^ None specified	Non-randomised clinical trial India Lactating mothers n=400	Started method after 6 weeks PP and took for 6 months: POP (desogestrel 75 µg)=200 Placebo=200	Infant outcomes Infant growth and development (‘normal’ defined as doubling birth weight by 4 months, tripled by 1 year; height 50 cm at birth increased to 62 cm at 6 months and 75 cm at 1 year; HC increased by 2 cm/month until 1 year; average and poor growth not defined).	Infant results Normal growth POP 99.5% vs placebo 98.5% Average growth POP 0.5% vs placebo 1% Poor growth POP 0% vs placebo 0.5% p=0.314	High
Kubba, 1966^24^ None specified	Cohort study Location not specified Women expressing a desire to lactate for 6 months or more n=90	Divided into 3 groups starting method at 4 weeks PP: POP (lynestrenol 5 mg then 2.5 mg after 2–3 weeks)=0 COC (lynesterol 2.5 mg, mestranol 0.075 mg)=30 Control (other means of contraception)=30	Breastfeeding outcomes Continuation (#) at 3 and 6 months Supplementation (#) at 3 and 6 months Infant outcomes Average weight (lbs) at 3 months and 6 months	Breastfeeding results Continuation: at 3 months, POP=23, COC=17, control=24; at 6 months, POP=21, COC=14, control=24 Supplementation: at 3 months, POP=10, COC=16, control=11; at 6 months, POP=13, COC=20, control=13 Infant results At 3 months, POP=13.8, COC=13.1, control=13.9; at 6 months, POP=16.11, COC=16.13, control=16.8	High
Parker, 2021^25^ National Institute of Nursing Research	Cohort study (secondary analysis of RCT) USA Mothers over the age of 18 years who delivered a very low birth weight infant n=170	Prior to hospital discharge: DMPA (mean 50 hours PP)=29 No DMPA=141	Breastfeeding outcomes Time to onset of secretory activation in hours (SE) – all participants, or excluding those with secretory activation prior to receiving DMPA Milk volume: g (SE) at days 1, 2, 3, 4, 5, 6, 7, 14, 21 Lactation until hospital discharge (%) (mean days to discharge: no DMPA=70 (34.6), DMPA=83.5 (30.8), p=0.026) Infant BM consumption: % of feeds (SE) at days 7 and 14	Breastfeeding results All: DMPA 118.0 (12.2), no DMPA 97.9 (5), p=0.133; exclusions: DMPA 129.9 (13.2), no DMPA 98.3 (4.9), p=0.028. Day 1 DMPA 1.1 (0.8), no DMPA 1.4 (0.4), p=0.703; day 2 DMPA 12.7 (3.4), no DMPA 9.9 (1.4), p=0.457; day 3 DMPA 58.3 (23.4), no DMPA 65.7 (9.7), p=0.773; day 4 DMPA 172.0 (36.3), no DMPA 166.0 (15.1), p=0.880; day 5 DMPA 316.7 (49.0), no DMPA 224.9 (20.5), p=0.090; day 6 DMPA 300.8 (51.1), no DMPA 253.5 (20.5), p=0.398; day 7 DMPA 358.5 (59.9), no DMPA 285.8 (24.3), p=0.268; day 14 DMPA 380.5 (56.4), no DMPA 284.0 (23.1), p=0.121; day 21 DMPA 328.6 (59.4), no DMPA 280.6 (23.2), p=0.457 DMPA 37.5%, no DMPA 47.5, p=0.387 Day 7: DMPA 75.9 (8.5), no DMPA 74.9 (3.5), p=0.908; Day 14: DMPA 81.4 (9.1), no DMPA 70.8 (3.7), p=0.287	High
Prema, 1982^26^ None specified	Cohort study India Lactating women n=2215	DMPA=22 NET-EN=51 COC1 (norgestrel/EE 150/50)=52 COC2 (norgestrel/EE 150/30)=50 Cu-IUD=68 Tubal ligation=55 No contraception=1917	Breastfeeding outcomes Mean duration of lactation in months (SD)	Breastfeeding results DMPA=22 (11.8) NET-EN=19 (10.9) COC1=21 (10.8) COC2=21 (11.0) Cu-IUD=21 (10.8) Tubal ligation=22 (10.7) No contraception=20 (9.6)	High
Sinchai, 1995^27^ N.V. Organon	RCT Thailand Healthy women with healthy infants at 6 weeks PP n=115	Randomised at 6 weeks PP: POP (lynestrenol, 500 µg)=62 IUD (Multiload Cu250)=53	Breastfeeding results Amount of BM produced from 20 min of pumping after waiting 3 hours at baseline, months 1, 3 and 6. Infant outcomes Infant weight (g) at baseline, 1, 3 and 6 months Infant length (cm) at baseline, 1, 3 and 6 months Infant biparietal HC (cm) at baseline, 1, 3 and 6 months Infants with illness in the previous month (%) at 1, 3 and 6 months	Breastfeeding results Baseline: POP=161 g, IUD=138 g (statistically significant difference); month 1: POP=156 g, IUD=173 g; month 3: POP=149 g, IUD=157 g; month 6, POP=137 g, IUD=142 g Infant results Baseline: POP=4410, IUD=4460; month 1: POP=5240, IUD=5260; month 3, POP=6350, IUD=6320; month 6, POP=7200, IUD=7170. Baseline: POP=54.5, IUD=55.3, p<0.05; month 1, POP=58.2, IUD=58.3; month 3, POP=62.7, IUD=62.4; month 6, POP=67.1, IUD=67.2 Baseline: POP=36.8, IUD=37.0; month 1, POP=38.5, IUD=38.5; month 3, POP=40.7, IUD=40.6; month 6, POP=42.8, IUD=48.2 Month 1, POP=27.6, IUD=15.4; month 3, POP=27.6, IUD=34.6; month 6, POP=42.1, IUD=75.0	High
Wongubol, 2010^28^ None specified	Non-randomised clinical trial Thailand Healthy women with normal birth weight infants at 6 weeks PP n=84	POP=42 IUD (Multiload Cu250)=42	Breastfeeding outcomes Average estimated 24-hour milk production (mL) at 10, 14, 18, 22, 26 and 30 weeks Infant outcomes Infant weight (g) at 10, 14, 18, 22, 26 and 30 weeks. Infant length (cm) 10, 14, 18, 22, 26 and 30 weeks Infant HC (cm) at 10, 14, 18, 22, 26 and 30 weeks Infant health: infection rates per 100	Breastfeeding results Estimated 24-hour milk production: 10 weeks, POP=2019.59, IUD=2294.88, p=0.20; 14 weeks, POP=1863.77, IUD=1931.34, p=0.83; 18 weeks, POP=1775.62, IUD=1823.12, p=0.74; 22 weeks, POP=1634.72, IUD=1626.71, p=0.72; 26 weeks, POP=1640.92, IUD=1646.05, p=0.75; 30 weeks, POP=1683.87, IUD=1557.44, p=0.96 Infant results 10 weeks, POP=5159.87, IUD=5338.29, p=0.14; 14 weeks, POP=5785.00, IUD=5916.59, p=0.32; 18 weeks, POP=6238.46, IUD=6371.10, p=0.36; 22 weeks, POP=6565.77, IUD=6731.95, p=0.29; 26 weeks, POP=6857.82, IUD=6970.49, p=0.49; 30 weeks, POP=7089.49, IUD=7185.61, p=0.57 10 weeks, POP=57.15, IUD=58.33, p=0.01; 14 weeks, POP=59.68, IUD=60.49, p=0.05; 18 weeks, POP=61.71, IUD=62.27, p=0.12; 22 weeks, POP=63.47, IUD=63.89, p=0.31; 26 weeks, POP=64.91, IUD=65.46, p=0.19; 30 weeks, POP=66.58, IUD=67.15, p=0.19 10 weeks, POP=38.48, IUD=38.65, p=0.44; 14 weeks, POP=39.66, IUD=39.78, p=0.58; 18 weeks, POP=40.62, IUD=40.72, p=0.63; 22 weeks, POP=41.46, IUD=41.48, p=0.95; 26 weeks, POP=42.31, IUD=42.11, p=0.40; 30 weeks, POP=42.85, IUD=42.79, p=0.80 10 weeks, POP=25.6, IUD=22.0, p=0.9; 14 weeks, POP=25.6 years, IUD=14.6, p=0.34; 18 weeks, POP=28.2, IUD=19.5, p=0.51; 22 weeks, POP=35.8, IUD=36.6, p=0.86; 26 weeks, POP=43.6, IUD=53.7, p=0.36; 30 weeks, POP=38.5, IUD=68.3, p=0.01	High

BF, breastfeeding; BM, breastmilk; COC, combined oral contraceptive; Cu, copper; DMPA, depot medroxyprogesterone acetate; EE, ethinylestradiol; HC, head circumference; IUD, intrauterine device; LNG, levonorgestrel; NET-EN, norethisterone enanthate; POP, progestogen-only pill; PP, postpartum; PVR, progesterone vaginal ring; RCT, randomised controlled trial; SD, standard deviation; SE, standard error.

One RCT had moderate RoB (online supplemental file 3) due to differential loss to follow-up and a lack of an intention-to-treat analysis.^20^ The remainder were assessed to have high RoB. One RCT did not adjust for differences between groups.^27^ Many cohort studies did not adequately describe methods (eg, response rates and/or loss to follow-up) or provided only crude estimates.^21^,^2628^

#### Breastfeeding outcomes

Seven articles reported on breastfeeding outcomes and reported on POPs,^24 27 28^ injectables^25 26^ and implants.^20 22^

##### Breastfeeding outcomes: POPs

A cohort study examined 90 women (location not reported) expressing a desire to lactate for at least 6 months. Participants were started at 4 weeks postpartum on a lynestrenol POP (n=30), a lynestrenol/mestranol combined oral contraceptive (COC) (n=30) or another contraceptive ‘control’ (n=30).^24^ At 3 months, the number of participants who continued breastfeeding was POP=23, COC=17, control=24; and at 6 months, POP=21, COC=14, control=24. At 3 months, the number of participants whose infants were supplemented with formula was POP=10, COC=16, control=11; and at 6 months, POP=13, COC=20, control=13. No statistical testing or information on follow-up was provided.

An RCT in Thailand randomised 115 breastfeeding women at 6 weeks postpartum to a lynestrenol POP or a Multiload Cu250 IUD.^27^ They reported the amount of breastmilk produced from 20 min of pumping and found a statistically significant increased amount of milk produced in the POP group (POP=161 g, IUD=138 g; no p-value) at baseline, but no differences at any timepoint during 6 months of follow-up. A non-randomised clinical trial enrolled 84 breastfeeding women in Thailand who initiated either a POP (n=42) or Multiload Cu250 IUD (n=42) at 6 weeks postpartum.^28^ They reported average estimated 24-hour milk production at 10, 14, 18, 22, 26 and 30 weeks and found no statistically significant differences between groups at any timepoint.

##### Breastfeeding outcomes: injectables

One cohort study in the USA included 170 breastfeeding mothers with a preterm, VLBW infant (≤1500 g and ≤32 weeks’ gestation). They compared those receiving depot medroxyprogesterone acetate (DMPA) prior to the mother’s hospital discharge (n=29, mean received at 50 hours postpartum) with those not receiving DMPA (n=141).^25^ The study found no differences in time to lactogenesis between groups (DMPA=118.0 hours (SE 12.2 hours), no-DMPA=97.9 hours (SE=5 hours); p=0.133). However, when excluding those who received DMPA after lactogenesis, there was an increased time to lactogenesis among the DMPA group (DMPA=129.9 hours (SE=13.2 hours), no-DMPA=98.3 hours (SE=4.9 hours); p=0.028). In the full cohort, they also found no differences in milk volume (measured at days 1–7, 14, 21, p>0.05 for each day) or in the percentage who continued lactation until hospital discharge (DMPA=37.5% vs no-DMPA=47.5%; p=0.387).

A cohort study in India of 2215 lactating women at an unspecified timing postpartum included those using DMPA (n=22), norethisterone enanthate (NET-EN; n=51), a norgestrel/ethinylestradiol (EE) 150/50 COC (COC1; n=52), a norgestrel/EE 150/30 COC (COC2; n=50), a Cu-IUD (n=68), tubal ligation (n=55) and no contraception (n=1917).^26^ The study reported mean duration of lactation for each group without statistical testing: DMPA=22 months (SD=11.8), NET-EN=19 months (SD=10.9), COC1=21 months (SD=10.8), COC2=21 months (11.0), Cu-IUD=21 months (SD=10.8), tubal ligation=22 months (SD=10.7) and no contraception=20 months (SD=9.6).

##### Breastfeeding outcomes: implants

An RCT in Brazil enrolled 24 breastfeeding women who were randomised within 48 hours postpartum to receive the etonogestrel (ETG) implant (n=12) or no method (n=12).^20^ At 6 weeks, the study found 92% of the implant group and 80% of the comparison group had continued breastfeeding (no statistical testing). They reported no differences in the mean amount of breastmilk ingested at days 29 (p=0.54) or 42 (p=0.63).

A non-randomised clinical trial enrolled 200 breastfeeding women in Chile who selected either a six-rod levonorgestrel (LNG) implant (n=100) or a TCu380A IUD (n=100) at 55 days postpartum.^22^ The study found the implant group had lower proportions of exclusive breastfeeding compared with the Cu-IUD (p<0.02) at month 12 and a higher frequency of supplementation with formula due to poor infant growth (no data provided).

### Infant outcomes

Six articles examined infant outcomes among healthy infants of mothers using POPs^21 23 24 27 28^ and implants.^20 22^

#### Infant outcomes: POPs

A non-randomised clinical trial in Mexico enrolled 231 breastfeeding women. At 1 week postpartum, participants selected either a lynestrenol POP (n=75), a Multiload Cu-IUD (n=76) or a non-hormonal non-IUD method for comparison (n=80).^21^ They reported infant mean weights and lengths monthly from months 1 to 6 without statistical testing. Mean weight for the POP group increased from 4194 g at month 1 to 8177 g at month 6; from 4086 g to 7996 g for the IUD group; and from 4069 g to 7604 g for the comparison group. The mean length for the POP group increased from 54.6 cm to 67.9 cm; from 54.1 cm to 67.8 cm for the IUD group; and from 53.6 cm to 66.9 cm for the comparison group.

A non-randomised clinical trial of 400 breastfeeding women in India received either a desogestrel POP (n=200) or placebo (n=200) at 6 weeks postpartum with continued use for 6 months.^23^ The study examined normal, average or poor infant growth (with “normal” defined as doubling birth weight by 4 months, triple by 1 year; height 50 cm at birth increased to 62 cm at 6 months and 75 cm at 1 year; head circumference increased by 2 cm/month until 1 year; average and poor growth not defined). They found no differences between groups (POP=99.5% normal growth, 0.5% average growth; placebo=98.5% normal, 1% average, 0.5% poor; p=0.314).

The cohort study of 90 women (location not reported) described above also reported average weight at 3 and 6 months without statistical testing.^24^ At 3 months, the average weight of the POP group was 13.8 lbs, COC=13.1 lbs and comparison=13.9 lbs; at 6 months, POP=16.11 lbs, COC=16.13 lbs and comparison=16.8 lbs. The RCT of 115 women in Thailand described above found no statistically significant differences in infant weight, length, head circumference and illness at baseline and months 1, 3 and 6 when comparing the POP and Cu-IUD groups.^27^ The non-randomised clinical trial of 84 women in Thailand described above also reported on infant weight, length, head circumference and infection rates at 10, 14, 18, 22, 26 and 30 weeks.^28^ At each timepoint, the means were similar between groups except for infant length at 10 weeks (POP=57.15 cm, IUD=58.33 cm; p=0.01) and 14 weeks (POP=59.68, IUD=60.49; p=0.05) and infection rates at 30 weeks (POP=38.5 per 100, IUD=68.3 per 100; p=0.01).

#### Infant outcomes: implants

The RCT of 24 women in Brazil described above found no differences in infant weight at 6 weeks (implant=4817 g (SD=534.9 g), comparison=4808 g (SD=653.7 g)); p=0.88).^20^ The non-randomised trial of 200 women in Chile described above found non-sustained decreased infant weight at 153 days (implant=7429 g (SD=705 g), IUD=7815 g (SD=812 g); p<0.05) and 183 days (implant=7978 g (SD=683 g), IUD=8345 g (SD=876 g); p<0.05), with no differences at other timepoints up to 1 year.^22^

### Summary of the evidence: Question 1

In total, 52 articles (9 newly identified described above and 43 included in the previous review) suggested no differences with POC use versus non-use in breastfeeding or infant outcomes.

Five new^21 23 24 27 28^ and 12 previously identified articles^14^ on POPs found no adverse effects on breastfeeding or infant outcomes. One previously identified article reported earlier supplementation among POP users (11.2 vs 15 weeks, no statistical testing) but normal infant growth parameters; one previously identified study (two articles) reported lower infant arm circumference but other normal growth parameters.^14^ One new^26^ and 14 previously identified articles^14^ on injectables reported no differences in breastfeeding or infant growth outcomes. One new article found a delay in lactogenesis among a subset of those using DMPA but no other differences in breastfeeding outcomes.^25^ One new^20^ and 16 previously identified articles (15 studies) found no sustained adverse effects of implant use on breastfeeding or infant outcomes; one previously identified study found a lower length increase among infants of mothers using an implant.^14^ One new article on the implant found lower rates of exclusive breastfeeding and higher rates of supplementation for infant growth concerns at 12 months compared with those using a Cu-IUD,^22^ though no numerical data were provided around these outcomes, so the clinical significance is unclear. No new studies included the LNG-IUD. Two previously identified articles on the LNG-IUD reported no differences in breastfeeding or infant outcomes. One previously identified study observed decreased breastfeeding at 8 months among LNG-IUD users but no differences in mean breastfeeding duration or infant growth.^14^

The CoE for most outcomes was very low, with two breastfeeding outcomes rated as low and one infant outcome as moderate.^31^ Concerns were primarily due to serious or very serious RoB, with some concerns regarding imprecision and inconsistency.

### Question 2: Among women who breastfeed, does the initiation of POC before 6 weeks postpartum increase the risk of poor breastfeeding or infant outcomes compared with the initiation of POC at 6 weeks or later?

Two newly identified articles addressed Question 2, assessing breastfeeding and infant outcomes among breastfeeding women who initiated POC before 6 weeks postpartum compared with those who initiated at 6 weeks or later (table 2).^29 30^ Both studied the implant, and one^30^ included both term and preterm infants. Both were from low- or middle-income countries.

**Table 2:** Characteristics of newly identified studies in a systematic review update on progestogen-only contraception (POC) use during breastfeeding addressing the research question ‘Among women who breastfeed, does the initiation of POC before 6 weeks postpartum increase the risk of poor breastfeeding or infant outcomes compared with the initiation of POC at 6 weeks or later?’

Author, year, funding	Study design, location, population	Interventions	Outcomes, follow-up duration	Results	Risk of bias
Averbach, 2019^30^ Society of Family Planning Research Fund, National Institutes of Health, Eunice Kennedy Shriver National Institute of Child Health & Human Development	RCT Uganda Women within 5 days PP n=205 randomised, 183 analysed (excluded multiples and perinatal mortality)	Implant (two-rod LNG) Randomised to implant (two-rod LNG): Immediate insertion (within 5 days PP)=96 Delayed insertion at 6–8 weeks PP=87	Breastfeeding outcomes Lactogenesis II: time to lactogenesis in hours (excluding those with lactogenesis prior to randomisation, immediate=55; delayed=42) BF continuation at 3 and 6 months Infant outcomes Infant weight gain (g) from birth to 6 months; sensitivity analysis among premature infants (defined as birth weight≤2500 g) HC increase (cm) from birth to 6 months Length increase (g) from birth to 6 months	Breastfeeding results Immediate=65 hours, IQR 48–79, range 26–128, delayed=63 hours, IQR 51–82, range 1–100, p=0.84 3 months, immediate=74%, delayed=71% delayed, p=0.74; 6 months, immediate=48% immediate, delayed=52%, p=0.58 Infant results Immediate=4632 g, delayed=4407 g, p=0.26 Premature: immediate=6033 g vs delayed=4563 g, p=0.006 Immediate=9.3 cm, delayed=9.5 cm, p=0.7 Immediate=14.7 cm, delayed=15.2 cm delayed, p=0.63	High (infant outcomes) Moderate (BF outcomes)
Carmo, 2017^29^ São Paulo Research Foundation, National Council for Scientific and Technological Development	RCT Brazil PP adult women and their infants n=100	Implant (etonogestrel) Randomised to early insertion within 48 hours PP=50 Conventional (6 weeks PP) insertion=50	Breastfeeding outcomes Exclusive BF rates at 14, 40, 90 and 180 days PP Infant outcomes Infant weight, mean kg (SD) at 14, 40, 90, 180, 270 and 360 days PP Infant height, mean cm (SD) at 14, 40, 90, 180, 270 and 360 days PP Infant HC, mean cm (SD) at 14, 40, 90, 180, 270 and 360 days PP Infant arm circumference, mean cm (SD) at 14, 40, 90, 180, 270 and 360 days PP	Breastfeeding results 14 days: PP=95.8%, 6 weeks=86%, p=0.16; 40 days: PP=87.8%, 6 weeks=76%, p=0.19; 90 days: PP=59.2%, 6 weeks=54%, p=0.69; 180 days: PP=2.2%, 6 weeks=8.2%, p=0.36 Infant results 14 days: PP=3.7 (0.5), 6 weeks=3.6 (0.4), p=0.56; 40 days: PP=4.8 (0.5), 6 weeks=4.7 (0.6), p=0.71; 90 days: PP=6.2 (0.7), 6 weeks=6.1 (0.8), p=0.46; 180 days: PP=8.0 (1.0), 6 weeks=7.9 (1.1), p=0.22; 270 days: PP=9.1 (1.0), 6 weeks=9.0 (1.2), p=0.37; 360 days: PP=10.1 (1.2), 6 weeks=9.8 (1.3), p=0.06 14 days: PP=52.0 (1.7), 6 weeks=51.5 (1.9), p=0.41; 40 days: PP=55.0 (2.1), 6 weeks=54.6 (2.9), p=0.40; 90 days: PP=60.5 (2.0), 6 weeks=60 (2.1), p=0.20; 180 days: PP=66.6 (2.1), 6 weeks=65.8 (2.1), p=0.05; 270 days: PP=71.4 (2.0), 6 weeks=70.8 (2.3), p=0.09; 360 days: PP=75.4 (2.1), 6 weeks=74.8 (2.6), p=0.09 14 days: PP=36.3 (1.1), 6 weeks=35.8 (1.3), p=0.05; 40 days: PP=38.1 (1.1), 6 weeks=37.8 (1.2), p=0.13; 90 days: PP=40.6 (1.1), 6 weeks=40.2 (1.1), p=0.08; 180 days: PP=43.2 (1.3), 6 weeks=43.0 (1.4), p=0.11; 270 days: PP=45.2 (1.3), 6 weeks=44.7 (1.5), p=0.02; 360 days: PP=46.4 (1.3), 6 weeks=46.1 (1.3), p=0.06 14 days: PP=10.8 (1.0), 6 weeks=10.8 (0.7), p=0.72; 40 days: PP=12.3 (1.0), 6 weeks=12.2 (1.0), p=0.90; 90 days: PP=13.6 (1.0), 6 weeks=13.6 (1.0), p=0.93; 180 days: PP=14.5 (1.1), 6 weeks=14.6 (1.2), p=1.0; 270 days: PP=15.2 (1.1), 6 weeks=15.1 (1.3), p=0.5; 360 days: PP=15.6 (1.0), 6 weeks=15.4 (1.3), p=0.17	High

BF, breastfeeding; HC, head circumference; IQR, interquartile range; IUD, intrauterine device; LNG, levonorgestrel; PP, postpartum; RCT, randomised controlled trial; SD, standard deviation.

One RCT had moderate RoB (online supplemental file 3) for breastfeeding outcomes due to missing data on the exposure; however, it had a high RoB for infant outcomes due to significant loss to follow-up.^30^ The other RCT had high RoB as it did not adjust for differences between groups.^29^

#### Breastfeeding and infant outcomes: implants

One RCT in Brazil enrolled 100 postpartum women who were randomised to early insertion of the ETG implant within 48 hours postpartum (n=50) or conventional insertion at 6 weeks postpartum (n=50).^29^ The study found no statistically significant differences in exclusive breastfeeding rates at 14, 40, 90 and 180 days postpartum between groups. They found no statistically significant differences in infant weight, height and arm circumference between groups at these same timepoints. At 270 days, infants in the early insertion group had greater mean head circumferences compared with conventional insertion (early=45.2 cm (SD 1.3 cm), conventional=44.7 cm (SD 1.5 cm); p=0.02), without differences at other timepoints.

An RCT in Uganda randomised 205 women within 5 days postpartum to immediate insertion of the two-rod LNG implant (n=96) or delayed insertion at 6–8 weeks postpartum (n=87).^30^ They found no differences in time to lactogenesis in the immediate versus delayed insertion groups (p=0.84) and no differences in breastfeeding continuation at 3 months (p=0.74) and 6 months (p=0.58). They also found no differences in infant weight gain (p=0.26), head circumference increase (p=0.7) or length increase (p=0.63) from birth to 6 months. A sensitivity analysis of “premature births” (defined by the authors as birth weight 2500 g, n not provided) found higher weight gain from birth to 6 months among those in the immediate insertion group compared with those in the delayed group (p=0.006).

#### Summary of the evidence: Question 2

Two newly identified^29 30^ and eight previously identified^14^ articles reported on early versus late initiation of POC. The two new studies^29 30^ suggest no differences in breastfeeding or infant outcomes with the initiation of POC before 6 weeks postpartum compared with initiation at 6 weeks or later, which is consistent with the eight studies previously reviewed for research Question 2.^14^ Both new studies found no negative effects of early initiation of the contraceptive implant on breastfeeding or infant outcomes, including among preterm infants. The prior review generally found no differences across multiple breastfeeding outcomes or infant growth, except for a cohort study and an RCT that found decreased rates of breastfeeding continuation with early initiation of implants and LNG-IUDs, respectively.^14^ The CoE was low or very low for all outcomes for Question 2, due primarily to serious or very serious RoB, and some concerns regarding imprecision and inconsistency.^31^

## Discussion

This updated review describes 11 newly identified articles on breastfeeding or infant outcomes with the use of POC while breastfeeding, which generally find no significant adverse effects on breastfeeding or infant outcomes. This is consistent with the prior review on this topic, while adding evidence on the use of DMPA and implants with preterm infants.

Strengths of this updated review include the comprehensive, updated search strategy and systematic approach. However, many studies had small numbers or were underpowered for our outcomes of interest. Many lacked methodological rigour, with poor reporting of or accounting for loss to follow-up, as well as a lack of accounting for confounders. When looking at the overall body of literature for our outcomes, the CoE is generally very low or low. All included articles had methodological concerns regarding RoB and limit the ability to draw firm conclusions, but continue to show no clear negative impacts on the use of POC among those who are breastfeeding.

This review did not include the progesterone vaginal ring (PVR), a contraceptive distinct from methods containing exogenous progestogens and designed for those who are postpartum and breastfeeding, and which may be considered when counselling about postpartum contraception if available.^32^ The PVR contains naturally occurring progesterone which, unlike synthetic progestogens, is inactivated if ingested orally and does not increase maternal serum levels of progesterone,^33^ so there is less theoretical concern with PVR use than POC use while breastfeeding. A separate systematic review in 2016 found no adverse effects of the PVR on breastfeeding or infant outcomes,^34^ and since then only one study has been published that continues to support the safety of the PVR during breastfeeding.^35^

Our findings are generally consistent with the prior review that found no negative effects from the use of POC while breastfeeding.^14^ This review adds some evidence on preterm infants, which may be helpful to support the counselling and care of this population which has a higher risk of breastfeeding difficulties.^36^ Further research among this population or others at risk of breastfeeding difficulties would help further clarify any potential risks.

## Supplementary material

10.1136/bmjsrh-2025-202837 online supplemental file 1

10.1136/bmjsrh-2025-202837 online supplemental file 2

10.1136/bmjsrh-2025-202837 online supplemental file 3

## Data Availability

All data relevant to the study are included in the article or uploaded as supplementary information.

## References

[1] World Health Organization (2022). WHO recommendations on maternal and newborn care for a positive postnatal experience. http://www.ncbi.nlm.nih.gov/books/NBK579657/.

[2] McKinney J, Keyser L, Clinton S (2018). ACOG Committee Opinion No. 736: Optimizing Postpartum Care. Obstet Gynecol.

[3] Meek JY, Noble L (2022). Policy Statement: Breastfeeding and the Use of Human Milk. Pediatrics.

[4] Labbok MH, Hight-Laukaran V, Peterson AE (1997). Multicenter study of the Lactational Amenorrhea Method (LAM): I. Efficacy, duration, and implications for clinical application. Contraception.

[5] Peterson AE, Peŕez-Escamilla R, Labbok MH (2000). Multicenter study of the lactational amenorrhea method (LAM) III: effectiveness, duration, and satisfaction with reduced client-provider contact. Contraception.

[6] Van der Wijden C, Manion C (2015). Lactational amenorrhoea method for family planning. Cochrane Database Syst Rev.

[7] Berens P, Labbok M, Breastfeeding M (2015). ABM Clinical Protocol #13: Contraception During Breastfeeding, Revised 2015. Breastfeed Med.

[8] Buhimschi CS (2004). Endocrinology of lactation. Obstet Gynecol Clin North Am.

[9] Kennedy KI, Short RV, Tully MR (1997). Premature introduction of progestin-only contraceptive methods during lactation. Contraception.

[10] Betrabet SS, Shikary ZK, Toddywalla VS (1987). ICMR Task Force Study on hormonal contraception. Transfer of norethisterone (NET) and levonorgestrel (LNG) from a single tablet into the infant’s circulation through the mother’s milk. Contraception.

[11] Croxatto HB, Díaz S (1987). The place of progesterone in human contraception. J Steroid Biochem.

[12] Quadros PS, Pfau JL, Wagner CK (2007). Distribution of progesterone receptor immunoreactivity in the fetal and neonatal rat forebrain. J Comp Neurol.

[13] González-Orozco JC, Camacho-Arroyo I (2019). Progesterone Actions During Central Nervous System Development. Front Neurosci.

[14] Phillips SJ, Tepper NK, Kapp N (2016). Progestogen-only contraceptive use among breastfeeding women: a systematic review. Contraception.

[15] World Health Organization (WHO) (2025). Medical eligibility criteria for contraceptive use, sixth edition.

[16] Page MJ, McKenzie JE, Bossuyt PM (2021). The PRISMA 2020 statement: an updated guideline for reporting systematic reviews. BMJ.

[17] https://www.covidence.org/.

[18] Sterne JAC, Savović J, Page MJ (2019). RoB 2: a revised tool for assessing risk of bias in randomised trials. BMJ.

[19] Guyatt G, Oxman AD, Akl EA (2011). GRADE guidelines: 1. Introduction-GRADE evidence profiles and summary of findings tables. J Clin Epidemiol.

[20] Braga GC, Ferriolli E, Quintana SM (2015). Immediate postpartum initiation of etonogestrel-releasing implant: A randomized controlled trial on breastfeeding impact. Contraception.

[R21] Delgado Betancourt J, Sandoval JC, Sanchez F (1984). Influence of Exluton (progestogen-only OC) and the Multiload Cu 250 IUD on lactation. Contracept Deliv Syst.

[22] Díaz S, Herreros C, Juez G (1985). Influence of Norplant contraceptive implants on lactation and infant growth. Rev Chil Obstet Ginecol.

[23] Dutta DK, Dutta I (2013). Desogestrel mini pill: is this safe in lactating mother?. J Indian Med Assoc.

[24] Kubba K (1966). The effect of oral progestagens on lactation. J Fac Med Baghdad.

[25] Parker LA, Sullivan S, Cacho N (2021). Effect of Postpartum Depo Medroxyprogesterone Acetate on Lactation in Mothers of Very Low-Birth-Weight Infants. Breastfeed Med.

[26] Prema K (1982). Duration of lactation and return of menstruation in lactating women using hormonal contraception and IUDs. Contracept Deliv Syst.

[27] Sinchai W, Sethavanich S, Asavapiriyanont S (1995). Effects of a progestogen-only pill (Exluton) and an intrauterine device (Multiload Cu250) on breastfeeding. Adv Contracept.

[28] Wongubol P (2010). The Different Effect of a Progestogen-only Pill and Intrauterine Device Contraception on Breast Milk Volume and Infant Growth. Reg 4-5 Med J.

[29] Carmo LS de MP, Braga GC, Ferriani RA (2017). Timing of Etonogestrel-Releasing Implants and Growth of Breastfed Infants: A Randomized Controlled Trial. Obstet Gynecol.

[30] Averbach S, Kakaire O, McDiehl R (2019). The effect of immediate postpartum levonorgestrel contraceptive implant use on breastfeeding and infant growth: a randomized controlled trial. Contraception.

[31] World Health Organization (WHO) (2025). Web Annex. Development of updated recommendations and Grading of Recommendations Assessment, Development and Evaluation (GRADE) tables. In: Medical eligibility criteria for contraceptive use, sixth edition.

[32] World Health Organization Department of Sexual and Reproductive Health and Research (WHO/SRH), Johns Hopkins Bloomberg School of Public Health, Johns Hopkins Center for Communication Programs (2022). Family Planning: A Global Handbook for Providers. 2022 Edition.

[33] RamaRao S, Clark H, Merkatz R (2013). Progesterone vaginal ring: introducing a contraceptive to meet the needs of breastfeeding women. Contraception.

[34] Carr SL, Gaffield ME, Dragoman MV (2016). Safety of the progesterone-releasing vaginal ring (PVR) among lactating women: A systematic review. Contraception.

[35] Roy M, Hazra A, Merkatz R (2020). Progesterone vaginal ring as a new contraceptive option for lactating mothers: Evidence from a multicenter non-randomized comparative clinical trial in India. Contraception.

[36] Boies EG, Vaucher YE, the Academy of Breastfeeding Medicine (2016). ABM Clinical Protocol #10: Breastfeeding the Late Preterm (34-36 6/7 Weeks of Gestation) and Early Term Infants (37-38 6/7 Weeks of Gestation), Second Revision 2016. Breastfeed Med.

