# Supplementary material for: Progestogen-only contraception use during breastfeeding: an updated systematic review
Source: BMJ Sex Reprod Health. 2025 Nov 3;51(Suppl 1):e202837. doi: 10.1136/bmjsrh-2025-202837 (PMC12703263; doi:10.1136/bmjsrh-2025-202837)
Supplement: online supplemental file 1 [file bmjsrh-51-Suppl_1-s001.docx]

**Supplementary File 1. Search strategy for systematic review on progestogen-only contraception use during breastfeeding.**

| **Database** | **Strategy** |
| --- | --- |
| **Medline**  **(OVID)**  **1946-** | 1. exp Progesterone/ OR exp Progestins/ 2. (Progestin* OR progesterone* OR progestogen* OR norethindrone OR medroxyprogesterone OR Norgestrel OR etonogestrel OR depo* OR provera OR hydroxyprogesterone* OR Levonorgestrel OR norethisterone OR nestorone* OR norprogesterone* OR gestagen* OR dmpa OR net-en OR minipill* OR mini-pill*).ti,ab,kf,hw. 3. 1 OR 2 4. Exp Contraceptive Agents, Female/ OR exp Contraceptives, Oral/ OR exp Intrauterine Devices, Medicated/ 5. (Contracept* OR birth control* OR patch OR pill* OR tablet* OR inject* OR ring* OR implant* OR intra-uterine OR intrauterine OR intravaginal OR intra-vaginal OR extra uterine OR extrauterine OR coil* OR patch* OR transdermal OR long acting OR post coital OR postcoital OR LNG-IUS OR IUD? OR IUS OR IUCD?).ti,ab,kf,hw. 6. 4 OR 5 7. 3 AND 6 8. (norplant* OR depo provera OR sayana press OR ortho evra OR xulane OR nuvaring OR mirena OR liletta OR kyleena OR Skyla OR implanon OR nexplanon OR jadelle OR uniplant OR sino-implant OR levoplant OR sinoplant OR cyclofem OR lunelle OR mesigyna OR cyclo provera OR cycloprovera).ti,ab,kf,hw. 9. 7 OR 8 10. exp Breast Feeding/ 11. (breast feed* OR breastfeed* OR breast fed OR breastfed OR lactat* OR breast milk* OR human milk OR bottle feed* OR bottle fed OR formula fed OR formula feed* OR wean*).ti,ab,hw,kf. 12. 10 OR 11 13. 9 AND 12 14. exp animals/ NOT exp humans/ 15. 13 NOT 14 |
| **Embase**  **(OVID)**  **1947-** | 1. exp Progesterone/ OR exp Gestagen/ 2. (Progestin* OR progesterone* OR progestogen* OR norethindrone OR medroxyprogesterone OR Norgestrel OR etonogestrel OR depo* OR provera OR hydroxyprogesterone* OR Levonorgestrel OR norethisterone OR nestorone* OR norprogesterone* OR gestagen* OR dmpa OR net-en OR minipill* OR mini-pill*).ti,ab,kf,hw. 3. 1 OR 2 4. Contraceptive Agent/ OR exp oral contraceptive agent/ OR exp intrauterine contraceptive device/ OR injectable contraceptive agent/ or postcoitus contraceptive agent/ 5. (Contracept* OR birth control* OR patch OR pill* OR tablet* OR inject* OR ring* OR implant* OR intra-uterine OR intrauterine OR intravaginal OR intra-vaginal OR extra uterine OR extrauterine OR coil* OR patch* OR transdermal OR long acting OR post coital OR postcoital OR LNG-IUS OR IUD? OR IUS OR IUCD?).ti,ab,kf,hw. 6. 4 OR 5 7. 3 AND 6 8. (norplant* OR depo provera OR sayana press OR ortho evra OR xulane OR nuvaring OR mirena OR liletta OR kyleena OR Skyla OR implanon OR nexplanon OR jadelle OR uniplant OR sino-implant OR levoplant OR sinoplant OR cyclofem OR lunelle OR mesigyna OR cyclo provera OR cycloprovera).ti,ab,kf,hw. 9. 7 OR 8 10. exp Breast Feeding/ 11. (breast feed* OR breastfeed* OR breast fed OR breastfed OR lactat* OR breast milk* OR human milk OR bottle feed* OR bottle fed OR formula fed OR formula feed* OR wean*).ti,ab,hw,kf. 12. 10 OR 11 13. 9 AND 12 14. exp animal/ NOT exp human/ 15. 13 NOT 14 16. Limit 15 to "remove medline records" 17. Limit 16 to conference abstract status 18. 16 not 17 |
| **CINAHL**  **(Ebsco)** | S1 (MH Progesterone+) OR (MH Progestins+)  S2 (Progestin* OR progesterone* OR progestogen* OR norethindrone OR medroxyprogesterone OR Norgestrel OR etonogestrel OR depo* OR provera OR hydroxyprogesterone* OR Levonorgestrel OR norethisterone OR nestorone* OR norprogesterone* OR gestagen* OR dmpa OR net-en OR minipill* OR mini-pill*)  S3 S1 OR S2  S4 ((MH "Contraceptive Agents, Female") OR (MH "Contraceptives, Oral") OR (MH "Intrauterine Devices, Medicated"))  S5 (Contracept* OR "birth control*" OR patch OR pill* OR tablet* OR inject* OR ring* OR implant* OR intra-uterine OR intrauterine OR intravaginal OR intra-vaginal OR "extra uterine" OR extrauterine OR coil* OR patch* OR transdermal OR "long acting" OR "post coital" OR postcoital OR LNG-IUS OR IUD? OR IUS OR IUCD?)  S6 S4 OR S5  S7 S3 AND S6  S8 (norplant* OR "depo provera" OR "sayana press" OR "ortho evra" OR xulane OR nuvaring OR mirena OR liletta OR kyleena OR Skyla OR implanon OR nexplanon OR jadelle OR uniplant OR sino-implant OR levoplant OR sinoplant OR cyclofem OR lunelle OR mesigyna OR "cyclo provera" OR cycloprovera)  S9 S7 OR S8  S10 (MH "Breast Feeding+")  S11 ("breast feed*" OR breastfeed* OR "breast fed" OR breastfed OR lactat* OR "breast milk*" OR "human milk" OR "bottle feed*" OR "bottle fed" OR "formula fed" OR "formula feed*" OR wean*)  S12 S10 OR S11  S13 S9 AND S12  Exclude Medline records |
| **Cochrane Library**  **(Cochrane Reviews and Cochrane Trials)** | #1 [mh ^Progesterone] OR [mh ^Progestins]  #2 (Progestin* OR progesterone* OR progestogen* OR norethindrone OR medroxyprogesterone OR Norgestrel OR etonogestrel OR depo* OR provera OR hydroxyprogesterone* OR Levonorgestrel OR norethisterone OR nestorone* OR norprogesterone* OR gestagen* OR dmpa OR "net-en" OR minipill* OR "mini-pill"):ti,ab,kw  #3 #1 OR #2  #4 [mh ^"Contraceptive Agents, Female"] OR [mh ^"Contraceptives, Oral"] OR [mh ^"Intrauterine Devices, Medicated"]  #5 (Contracept* OR "birth control" OR patch OR pill* OR tablet* OR inject* OR ring* OR implant* OR intra-uterine OR intrauterine OR intravaginal OR intra-vaginal OR "extra uterine" OR extrauterine OR coil* OR patch* OR transdermal OR "long acting" OR "post coital" OR postcoital OR LNG-IUS OR IUD? OR IUS OR IUCD?):ti,ab,kw  #6 #4 OR #5  #7 #3 AND #6  #8 (norplant* OR "depo provera" OR "sayana press" OR "ortho evra" OR xulane OR nuvaring OR mirena OR liletta OR kyleena OR Skyla OR implanon OR nexplanon OR jadelle OR uniplant OR sino-implant OR levoplant OR sinoplant OR cyclofem OR lunelle OR mesigyna OR "cyclo provera" OR cycloprovera)  #9 #7 OR #8  #10 [mh ^"breast feeding"]  #11 ("breast feed" OR "breast feeding" OR breastfeed* OR "breast fed" OR breastfed OR lactat* OR "breast milk" OR "human milk" OR "bottle feed" OR "bottle feeding" OR "bottle fed" OR "formula fed" OR "formula feed" OR "formula feeding" OR wean*):ti,ab,kw  #12 #10 OR #11  #13 #9 AND #12 |
| **Clinicaltrials.gov** | Progestin OR progestins OR progesterone OR norethindrone OR medroxyprogesterone Hydroxyprogesterone OR Levonorgestrel OR norethisterone OR nestorone OR norprogesterone OR gestagen OR nestorones OR norprogesterones OR gestagens OR minipill OR mini-pill\| breast feed OR breastfeed breastfeeding OR breast feeding OR lactation OR lactating OR breast milk OR bottle feed OR bottle feeding OR bottle fed OR formula fed OR formula feed OR formula feeding OR wean OR weaning  OR  Contraceptive OR contraceptives OR contraception OR birth control \| breast feed OR breastfeed breastfeeding OR breast feeding OR lactation OR lactating OR breast milk OR bottle feed OR bottle feeding OR bottle fed OR formula fed OR formula feed OR formula feeding OR wean OR weaning  OR  norplant* OR depo provera OR sayana press OR ortho evra OR xulane OR nuvaring OR mirena OR liletta OR kyleena OR Skyla OR implanon OR nexplanon OR jadelle OR uniplant OR sino-implant OR levoplant OR sinoplant OR cyclofem OR lunelle OR mesigyna OR cyclo provera OR cycloprovera\| breast feed OR breastfeed breastfeeding OR breast feeding OR lactation OR lactating OR breast milk OR bottle feed OR bottle feeding OR bottle fed OR formula fed OR formula feed OR formula feeding OR wean OR weaning |
