# Supplementary material for: Progestogen-only contraception use during breastfeeding: an updated systematic review
Source: BMJ Sex Reprod Health. 2025 Nov 3;51(Suppl 1):e202837. doi: 10.1136/bmjsrh-2025-202837 (PMC12703263; doi:10.1136/bmjsrh-2025-202837)
Supplement: online supplemental file 2 [file bmjsrh-51-Suppl_1-s002.docx]

Supplementary File 2. Complete line listing of articles meeting inclusion criteria for systematic review on progestogen-only contraception use during breastfeeding.

| Research question 1: among women who breastfeed, does the use of POC increase the risk of poor breastfeeding or infant outcomes compared with those who do not use POC? |
| --- |

| **Lead Author(s)** | **Year** | **Newly identified for 2025 review** | **Study design** | **Contraceptive method** | **Comparison** | **Healthy or at risk population*** | **Timing** | **Outcomes** |
| --- | --- | --- | --- | --- | --- | --- | --- | --- |
| Kubba^1^ | 1966 | Yes | Cohort | POP | Non-hormonal | Healthy | < 6 weeks | Breastfeeding  Infant |
| Kamal^2^ | 1969 | No | NRCT | POP | COC, non-hormonal | Healthy | > 6 weeks | Breastfeeding  Infant |
| Kamal^3^ | 1970 | No | NRCT | POP | COC, EE, non-hormonal | Healthy | < 6 weeks | Breastfeeding  Infant |
| Karim^4^ | 1971 | No | Cohort | Injectables | Non-hormonal | Healthy | < 6 weeks | Breastfeeding  Infant |
| Guiloff^5^ | 1974 | No | Cohort | POP, Injectables | COC, non-hormonal | Healthy | < 6 weeks | Breastfeeding |
| Giner Velasquez^6^ | 1976 | No | RCT | Injectables | Non-hormonal | Healthy | < 6 weeks | Breastfeeding  Infant |
| Zañartu^7^ | 1976 | No | Cohort | Injectables | Non-hormonal | Healthy | < 6 weeks | Breastfeeding |
| Zañartu^8^ | 1976 | No | NRCT | POP | Non-hormonal | Healthy | Mixed | Breastfeeding |
| Seth^9^ | 1977 | No | Cohort | Implant | Non-hormonal | Healthy | Mixed | Breastfeeding  Infant |
| Prema^10^ | 1982 | Yes | Cohort | Injectables | COC, non-hormonal | Healthy | > 6 weeks | Breastfeeding |
| Croxatto^11^ | 1982 | No | Cohort | Progesterone pellets | Non-hormonal | Healthy | < 6 weeks | Breastfeeding  Infant |
| Dahlberg^12^ | 1982 | No | Cohort | Injectables | Non-hormonal | Healthy | < 6 weeks | Infant |
| Heikkila^13^ | 1982 | No | RCT | LNG IUD | Non-hormonal | Healthy | < 6 weeks | Breastfeeding  Infant |
| West^14^ | 1983 | No | Cohort | POP | COC, non-hormonal | Healthy | < 6 weeks | Breastfeeding |
| Delgado Betancourt^15^ | 1984 | Yes | NRCT | POP | Non-hormonal | Healthy | < 6 weeks | Infant |
| Diaz^16^ | 1984 | No | Cohort | Progesterone pellets | Non-hormonal | Healthy | < 6 weeks | Breastfeeding  Infant |
| Jimenez^17^ | 1984 | No | Cohort | Injectables | Non-hormonal | Healthy | < 6 weeks | Breastfeeding  Infant |
| Tankeyoon^18^ | 1984 | No | Cohort | POP, Injectables | Non-hormonal | Healthy | > 6 weeks | Breastfeeding  Infant |
| Diaz^19^ | 1985 | Yes | NRCT | Implant | Non-hormonal | Healthy | > 6 weeks | Breastfeeding  Infant |
| Abdulla^20^ | 1985 | No | Cohort | Implant | Non-hormonal | Healthy | < 6 weeks | Infant |
| Shaaban^21^ | 1985 | No | Cohort | Implant | Non-hormonal | Healthy | < 6 weeks | Breastfeeding  Infant |
| Shikary^22^ | 1986 | No | Cohort | POP, implant | Non-hormonal | Healthy | < 6 weeks | Infant |
| Zacharias^23^ | 1986 | No | Cohort | POP, Injectables | Non-hormonal | Healthy | < 6 weeks | Breastfeeding |
| Affandi^24^ | 1986 | No | Cohort | Implant | Non-hormonal | Healthy | < 6 weeks | Infant |
| McCann^25^ | 1989 | No | Cohort | POP | Non-hormonal | Healthy | < 6 weeks | Breastfeeding  Infant |
| Moggia^26^ | 1991 | No | Cohort | POP | Non-hormonal | Healthy | < 6 weeks | Breastfeeding  Infant |
| Shaaban^27^ | 1991 | No | Cohort | Injectables, implant | Non-hormonal | Healthy | < 6 weeks | Breastfeeding  Infant |
| Pardthaisong^28^ | 1992 | No | Cohort | Injectables | Non-hormonal | Healthy | < 6 weeks | Infant |
| WHO^29^   WHO^30^ | 1994   1994 | No | Cohort | POP, Injectables, implant | Non-hormonal | Healthy | > 6 weeks | Breastfeeding  Infant |
| Sinchai^31^ | 1995 | Yes | RCT | POP | Non-hormonal | Healthy | > 6 weeks | Breastfeeding  Infant |
| Abdel-Aleem^32^ | 1996 | No | Cohort | Implant | Non-hormonal | Healthy | < 6 weeks | Breastfeeding  Infant |
| Hannon^33^ | 1997 | No | Cohort | Injectables | Non-hormonal | Healthy | < 6 weeks | Breastfeeding |
| Diaz^34^ | 1997 | No | Cohort | POP, Implant | Non-hormonal | Healthy | > 6 weeks | Breastfeeding  Infant |
| Lawrie^35^ | 1998 | No | RCT | Injectables | Non-hormonal | Healthy | < 6 weeks | Breastfeeding |
| Coutinho^36^ | 1999 | No | Cohort | Implant | Non-hormonal | Healthy | > 6 weeks | Breastfeeding  Infant |
| Diaz^37^ | 1999 | No | Cohort | Implant | Non-hormonal | Healthy | > 6 weeks | Breastfeeding  Infant |
| Bjarnadottir^38^ | 2001 | No | Cohort | POP | Non-hormonal | Healthy | < 6 weeks | Breastfeeding  Infant |
| Baheiraei^39^ | 2001 | No | Cohort | POP, Injectables | Non-hormonal | Healthy | > 6 weeks | Infant |
| Massai^40^ | 2001 | No | Cohort | Implant | Non-hormonal | Healthy | > 6 weeks | Breastfeeding  Infant |
| Halderman^41^ | 2002 | No | Cohort | POP, Injectables, implant | Non-hormonal | Healthy | < 6 weeks | Breastfeeding |
| Schiappacasse^42^   Diaz^43^ | 2002   1985 | No | Cohort | Implant | Non-hormonal | Healthy | > 6 weeks | Breastfeeding  Infant |
| Shaamash^44^ | 2005 | No | RCT | LNG-IUD | Non-hormonal | Healthy | > 6 weeks | Breastfeeding  Infant |
| Taneepanichskul^45^   Reinprayoon^46^ | 2006   2000 | No | Cohort | Implant | Non-hormonal | Healthy | < 6 weeks | Breastfeeding  Infant |
| Wongubol^47^ | 2010 | Yes | NRCT | POP | Non-hormonal | Healthy | > 6 weeks | Breastfeeding  Infant |
| Costa^48^ | 2012 | No | Cohort | POP, Injectables, LNG-IUD | Non-hormonal | Healthy | > 6 weeks | Breastfeeding |
| Espey^49^ | 2012 | No | RCT | POP | COC | Healthy | < 6 weeks | Breastfeeding  Infant |
| Dutta^50^ | 2013 | Yes | NRCT | POP | Non-hormonal | Healthy | > 6 weeks | Infant |
| Brownell^51^ | 2013 | No | Cohort | Injectables | Non-hormonal | Healthy | < 6 weeks | Breastfeeding |
| Bahamondes^52^ | 2013 | No | Cohort | Implant, LNG-IUD | COC, non-hormonal | Healthy | > 6 weeks | Breastfeeding  Infant |
| Singhal^53^ | 2014 | No | Cohort | Injectables | Non-hormonal | Healthy | < 6 weeks | Breastfeeding  Infant |
| Braga^54^ | 2015 | Yes | RCT | Implant | Non-hormonal | Healthy | < 6 weeks | Breastfeeding  Infant |
| Parker^55^ | 2021 | Yes | Cohort | Injectables | Non-hormonal | At risk | < 6 weeks | Breastfeeding |

*At risk for breastfeeding difficulties

| Research question 2: among women who breastfeed, does the initiation of progestogen-only contraception before 6 weeks postpartum increase the risk of adverse breastfeeding or infant outcomes compared with the initiation of progestogen-only contraception at 6 weeks or later? |
| --- |

| Lead Author(s) | Year | Newly identified for 2025 review | Study design | Contraceptive method | Comparison | Healthy or at risk population* | Outcomes |
| --- | --- | --- | --- | --- | --- | --- | --- |
| Karim^4^ | 1971 | No | Cohort | Injectables | 7 days vs 42 days postpartum | Healthy | Infant |
| Seth^9^ | 1977 | No | Cohort | Implant | 6 days vs 6 weeks postpartum | Healthy | Breastfeeding  Infant |
| Dahlberg^12^ | 1982 | No | Cohort | Injectables | Within 48 hours postpartum vs later | Healthy | Infant |
| Diaz^16^ | 1984 | No | Cohort | Progesterone pellets | 30 days vs 60 days postpartum | Healthy | Breastfeeding  Infant |
| Brito^56^ | 2009 | No | RCT | Implant (early) vs Injectible (delayed) | 24-48 hrs vs 6 weeks postpartum | Healthy | Breastfeeding |
| Chen^57^ | 2011 | No | RCT | LNG-IUD | Immediate post-placental vs 6-8 weeks postpartum | Healthy | Breastfeeding |
| Gurtcheff^58^ | 2011 | No | RCT | Implant | 1-3 days vs 4-8 weeks postpartum | Healthy | Breastfeeding |
| Matias^59^ | 2012 | No | Cohort | Injectables | By 72 hrs vs 1 month vs 3 months vs 6 months postpartum | Healthy | Breastfeeding |
| Carmo^60^ | 2017 | Yes | RCT | Implant | Within 48 hours vs 6 weeks postpartum | Healthy | Breastfeeding  Infant |
| Averbach^61^ | 2019 | Yes | RCT | Implant | Within 5 days vs 6-8 weeks postpartum | Mixed | Breastfeeding  Infant |

*At risk for breastfeeding difficulties

Abbreviations: COC = combined oral contraceptive, EE = ethinyl estradiol, IUD = intrauterine device, LNG = levonorgestrel, NRCT = non randomized clinical trial, POC = progestogen-only contraception, POP = progestogen-only pill, RCT = randomized controlled trial

References

1. Kubba K. The effect of oral progestagens on lactation. *J*. 1966;Fac. Med. 8(2):66-69.

2. Kamal I, Hefnawi F, Ghoneim M, et al. Clinical, biochemical, and experimental studies on lactation. II. Clinical effects of gestagens on lactation. *Am J Obstet Gynecol*. 1969;105(3):324-334.

3. Kamal I, Hefnawi F, Ghoneim M. Clinical, biochemical, and experimental studies on lactation. V. Clinical effects of steroids on the initiation of lactation. *Am J Obstet Gynecol Print*. 1970;108(4):655-658.

4. Karim M, Ammar R, el-Mahgoub S, el-Ganzoury B, Fikri F, Abdou I. Injected progestogen and lactation. *Br Med J*. 1971;1(5742):200-203.

5. Guiloff E, Ibarra-Polo A, Zanartu J, Toscanini C, Mischler TW, Gomez-Rogers C. Effect of contraception on lactation. *Am J Obstet Gynecol*. 1974;118(1):42-45.

6. Giner Velazquez J, Cortes Gallegos V, Sotelo Lopez A, Bondani G. [Effect of daily oral administration of 0.350 mg of norethindrone on lactation and on the composition of milk]. *Ginecol Obstet Mex*. 1976;40(237):31-39.

7. Zanartu J, Aguilera E, Munoz G, Peliowsky H. Effect of a long-acting contraceptive progestogen on lactation. *Obstet Gynecol*. 1976;47(2):174-176.

8. Zanartu J, Aguilera E, Munoz-Pinto G. Maintenance of lactation by means of continuous low-dose progestogen given post-partum as a contraceptive. *Contraception*. 1976;13(3):313-318.

9. Seth U, Yadava HS, Agarwal N, Laumas KR, Hingorani V. Effect of a subdermal silastic implant containing norethindrone acetate on human lactation. *Contraception*. 1977;16(4):383-398.

10. Prema K. Duration of lactation and return of menstruation in lactating women using hormonal contraception and IUDs. *Contracept Deliv Syst*. 1982;3(1):39-46.

11. Croxatto HB, Diaz S, Peralta O, et al. Fertility regulation in nursing women. II. Comparative performance of progesterone implants versus placebo and copper T. *Am J Obstet Gynecol*. 1982;144(2):201-208.

12. Dahlberg K. Some effects of depo-medroxyprogesterone acetate (DMPA): observations in the nursing infant and in the long-term user. *Int J Gynaecol Obstet*. 1982;20(1):43-48.

13. Heikkila M, Luukkainen T. Duration of breast-feeding and development of children after insertion of a levonorgestrel-releasing intrauterine contraceptive device. *Contraception*. 1982;25(3):279-292.

14. West CP. The acceptability of a progestagen-only contraceptive during breast-feeding. *Contraception*. 1983;27(6):563-569.

15. Delgado Betancourt J, Sandoval JC, Sanchez F, Vallesteros De Cano P, De La Luz Bantista M, Jimenez F. Influence of Exluton (progestogen-only OC) and the Multiload Cu 250 IUD on lactation. *Contracept Deliv Syst*. 1984;5(2):91-95.

16. Diaz S, Peralta O, Juez G, et al. Fertility regulation in nursing women. VI. Contraceptive effectiveness of a subdermal progesterone implant. *Contraception*. 1984;30(4):311-325.

17. Jimenez J, Ochoa M, Soler MP, Portales P. Long-term follow-up of children breast-fed by mothers receiving depot-medroxyprogesterone acetate. *Contraception*. 1984;30(6):523-533.

18. Tankeyoon M, Dusitsin N, Chalapati S, et al. Effects of hormonal contraceptives on milk volume and infant growth. WHO Special Programme of Research, Development and Research Training in Human Reproduction Task force on oral contraceptives. *Contraception*. 1984;30(6):505-522.

19. Diaz S, Herreros C, Juez G, Peralta O, Croxatto HB. [Influence of Norplant contraceptive implants on lactation and infant growth]. *Rev Chil Obstet Ginecol*. 1985;50(5):421-428.

20. Abdulla KA, Elwan SI, Salem HS, Shaaban MM. Effect of early postpartum use of the contraceptive implants, NORPLANT, on the serum levels of immunoglobulins of the mothers and their breastfed infants. *Contraception*. 1985;32(3):261-266.

21. Shaaban MM, Salem HT, Abdullah KA. Influence of levonorgestrel contraceptive implants, NORPLANT, initiated early postpartum upon lactation and infant growth. *Contraception*. 1985;32(6):623-635.

22. Shikary ZK, Betrabet SS, Toddywala WS, Patel DM, Datey S, Saxena BN. Pharmacodynamic effects of levonorgestrel (LNG) administered either orally or subdermally to early postpartum lactating mothers on the urinary levels of follicle stimulating hormone (FSH), luteinizing hormone (LH) and testosterone (T) in their breast-fed male infants. *Contraception*. 1986;34(4):403-412.

23. Zacharias S, Aguilera E, Assenzo JR, Zanartu J. Effects of hormonal and nonhormonal contraceptives on lactation and incidence of pregnancy. *Contraception*. 1986;33(3):203-213.

24. Affandi B, Karmadibrata S, Prihartono J, Lubis F, Samil RS. Effect of Norplant on mothers and infants in the postpartum period. *Adv Contracept*. 1986;2(4):371-380.

25. McCann MF, Moggia AV, Higgins JE, Potts M, Becker C. The effects of a progestin-only oral contraceptive (levonorgestrel 0.03 mg) on breast-feeding. *Contraception*. 1989;40(6):635-648.

26. Moggia AV, Harris GS, Dunson TR, et al. A comparative study of a progestin-only oral contraceptive versus non-hormonal methods in lactating women in Buenos Aires, Argentina. *Contraception*. 1991;44(1):31-43.

27. Shaaban MM. Contraception with progestogens and progesterone during lactation. *J Steroid Biochem Mol Biol*. 1991;40(4-6):705-710.

28. Pardthaisong T, Yenchit C, Gray R. The long-term growth and development of children exposed to Depo-Provera during pregnancy or lactation. *Contraception*. 1992;45(4):313-324.

29. Anonymous. Progestogen-only contraceptives during lactation: I. Infant growth. World Health Organization Task force for Epidemiological Research on Reproductive Health; Special Programme of Research, Development and Research Training in Human Reproduction. *Contraception*. 1994;50(1):35-53.

30. Anonymous. Progestogen-only contraceptives during lactation: II. Infant development. World Health Organization, Task Force for Epidemiological Research on Reproductive Health; Special Programme of Research, Development, and Research Training in Human Reproduction. *Contraception*. 1994;50(1):55-68.

31. Sinchai W, Sethavanich S, Asavapiriyanont S, et al. Effects of a progestogen-only pill (Exluton) and an intrauterine device (Multiload Cu250) on breastfeeding. *Adv Contracept*. 1995;11(2):143-155.

32. Abdel-Aleem H, Abol-Oyoun el SM, Shaaban MM, et al. The use of nomegestrol acetate subdermal contraceptive implant, uniplant, during lactation. *Contraception*. 1996;54(5):281-286.

33. Hannon PR, Duggan AK, Serwint JR, Vogelhut JW, Witter F, DeAngelis C. The influence of medroxyprogesterone on the duration of breast-feeding in mothers in an urban community. *Arch Pediatr Adolesc Med*. 1997;151(5):490-496.

34. Diaz S, Zepeda A, Maturana X, et al. Fertility regulation in nursing women. IX. Contraceptive performance, duration of lactation, infant growth, and bleeding patterns during use of progesterone vaginal rings, progestin-only pills, Norplant implants, and Copper T 380-A intrauterine devices. *Contraception*. 1997;56(4):223-232.

35. Lawrie TA, Hofmeyr GJ, De Jager M, Berk M, Paiker J, Viljoen E. A double-blind randomised placebo controlled trial of postnatal norethisterone enanthate: the effect on postnatal depression and serum hormones. *Br J Obstet Gynaecol*. 1998;105(10):1082-1090.

36. Coutinho EM, Athayde C, Dantas C, Hirsch C, Barbosa I. Use of a single implant of elcometrine (ST-1435), a nonorally active progestin, as a long acting contraceptive for postpartum nursing women. *Contraception*. 1999;59(2):115-122.

37. Diaz S, Reyes MV, Zepeda A, et al. Norplant((R)) implants and progesterone vaginal rings do not affect maternal bone turnover and density during lactation and after weaning. *Hum Reprod*. 1999;14(10):2499-2505.

38. Bjarnadottir RI, Gottfredsdottir H, Sigurdardottir K, Geirsson RT, Dieben TO. Comparative study of the effects of a progestogen-only pill containing desogestrel and an intrauterine contraceptive device in lactating women. *BJOG Int J Obstet Gynaecol*. 2001;108(11):1174-1180.

39. Baheiraei A, Ardsetani N, Ghazizadeh S. Effects of progestogen-only contraceptives on breast-feeding and infant growth. *Int J Gynaecol Obstet*. 2001;74(2):203-205.

40. Massai MR, Diaz S, Quinteros E, et al. Contraceptive efficacy and clinical performance of Nestorone implants in postpartum women. *Contraception*. 2001;64(6):369-376.

41. Halderman LD, Nelson AL. Impact of early postpartum administration of progestin-only hormonal contraceptives compared with nonhormonal contraceptives on short-term breast-feeding patterns. *Am J Obstet Gynecol*. 2002;186(6):1250-1256; discussion 1256.

42. Schiappacasse V, Diaz S, Zepeda A, Alvarado R, Herreros C. Health and growth of infants breastfed by Norplant contraceptive implants users: a six-year follow-up study. *Contraception*. 2002;66(1):57-65.

43. Diaz S, Herreros C, Juez G, et al. Fertility regulation in nursing women: VII. Influence of NORPLANT levonorgestrel implants upon lactation and infant growth. *Contraception*. 1985;32(1):53-74.

44. Shaamash AH, Sayed GH, Hussien MM, Shaaban MM. A comparative study of the levonorgestrel-releasing intrauterine system Mirena versus the Copper T380A intrauterine device during lactation: breast-feeding performance, infant growth and infant development. *Contraception*. 2005;72(5):346-351.

45. Taneepanichskul S, Reinprayoon D, Thaithumyanon P, Praisuwanna P, Tosukhowong P, Dieben T. Effects of the etonogestrel-releasing implant Implanon and a nonmedicated intrauterine device on the growth of breast-fed infants. *Contraception*. 2006;73(4):368-371.

46. Reinprayoon D, Taneepanichskul S, Bunyavejchevin S, et al. Effects of the etonogestrel-releasing contraceptive implant (Implanon on parameters of breastfeeding compared to those of an intrauterine device. *Contraception*. 2000;62(5):239-246.

47. Wongubol P. The Different Effect of a Progestogen-only Pill and Intrauterine Device Contraception on Breast Milk Volume and Infant Growth. *Reg 4-5 Med J วารสารแพทย์เขต 4-5*. 2010;29(3):303‐314.

48. Costa ML, Cecatti JG, Krupa FG, Rehder PM, Sousa MH, Costa-Paiva L. Progestin-only contraception prevents bone loss in postpartum breastfeeding women. *Contraception*. 2012;85(4):374-380. doi:10.1016/j.contraception.2011.08.015

49. Espey E, Ogburn T, Leeman L, Singh R, Ostrom K, Schrader R. Effect of progestin compared with combined oral contraceptive pills on lactation: a randomized controlled trial. *Obstet Gynecol*. 2012;119(1):5-13. doi:10.1097/AOG.0b013e31823dc015

50. Dutta DK, Dutta I. Desogestrel mini pill: is this safe in lactating mother? *J Indian Med Assoc*. 2013;111(8):553-555.

51. Brownell EA, Fernandez ID, Fisher SG, et al. The effect of immediate postpartum depot medroxyprogesterone on early breastfeeding cessation. *Contraception*. 2013;87(6):836-843. doi:10.1016/j.contraception.2012.08.045

52. Bahamondes L, Bahamondes MV, Modesto W, et al. Effect of hormonal contraceptives during breastfeeding on infant’s milk ingestion and growth. *Fertil Steril*. 2013;100(2):445-450. doi:10.1016/j.fertnstert.2013.03.039

53. Singhal S, Sarda N, Gupta S, Goel S. Impact of injectable progestogen contraception in early puerperium on lactation and infant health. *J Clin Diagn Res JCDR*. 2014;8(3):69-72. doi:10.7860/JCDR/2014/7775.4110

54. Braga GC, Ferriolli E, Quintana SM, Ferriani RA, Pfrimer K, Vieira CS. Immediate postpartum initiation of etonogestrel-releasing implant: A randomized controlled trial on breastfeeding impact. *Contraception*. 2015;92(6):536-542. doi:10.1016/j.contraception.2015.07.009

55. Parker LA, Sullivan S, Cacho N, Krueger C, Mueller M. Effect of Postpartum Depo Medroxyprogesterone Acetate on Lactation in Mothers of Very Low-Birth-Weight Infants. *Breastfeed Med Off J Acad Breastfeed Med*. 2021;16(10):835-842. doi:10.1089/bfm.2020.0336

56. Brito MB, Ferriani RA, Quintana SM, Yazlle ME, Silva de Sa MF, Vieira CS. Safety of the etonogestrel-releasing implant during the immediate postpartum period: a pilot study. *Contraception*. 2009;80(6):519-526. doi:10.1016/j.contraception.2009.05.124

57. Chen BA, Reeves MF, Creinin MD, Schwarz EB. Postplacental or delayed levonorgestrel intrauterine device insertion and breast-feeding duration. *Contraception*. 2011;84(5):499-504. doi:10.1016/j.contraception.2011.01.022

58. Gurtcheff SE, Turok DK, Stoddard G, Murphy PA, Gibson M, Jones KP. Lactogenesis after early postpartum use of the contraceptive implant: a randomized controlled trial. *Obstet Gynecol*. 2011;117(5):1114-1121. doi:10.1097/AOG.0b013e3182165ee8

59. Matias SL, Nommsen-Rivers LA, Dewey KG. Determinants of exclusive breastfeeding in a cohort of primiparous periurban peruvian mothers. *J Hum Lact*. 2012;28(1):45-54. doi:10.1177/0890334411422703

60. Carmo L, Braga GC, Ferriani RA, Quintana SM, Vieira CS. Timing of Etonogestrel-Releasing Implants and Growth of Breastfed Infants: A Randomized Controlled Trial. *Obstet Gynecol*. 2017;130(1):100-107. doi:10.1097/AOG.0000000000002092

61. Averbach S, Kakaire O, McDiehl R, Dehlendorf C, Lester F, Steinauer J. The effect of immediate postpartum levonorgestrel contraceptive implant use on breastfeeding and infant growth: a randomized controlled trial. *Contraception*. 2019;99(2):87-93. doi:10.1016/j.contraception.2018.10.008
