# Supplementary material for: Progestogen-only contraception use during breastfeeding: an updated systematic review
Source: BMJ Sex Reprod Health. 2025 Nov 3;51(Suppl 1):e202837. doi: 10.1136/bmjsrh-2025-202837 (PMC12703263; doi:10.1136/bmjsrh-2025-202837)
Supplement: online supplemental file 3 [file bmjsrh-51-Suppl_1-s003.docx]

**Supplementary file 3. Risk of bias assessments for an updated systematic review on the safety of intrauterine devices during breastfeeding**

| **Study** | **Randomization** | **Deviations** | **Missing Data** | **Measurement** | **Reporting** | **Overall risk of bias** |
| --- | --- | --- | --- | --- | --- | --- |
| *Question 1- Randomized controlled trials* | | | | | | |
| Sinchai 1995^1^ |  |  |  |  |  |  |
| Braga 2015^2^ |  |  |  |  |  |  |

| **Study** | **Selection bias** | **Information bias** | **Confounding** | **Overall risk of bias** |  |  |
| --- | --- | --- | --- | --- | --- | --- |
| *Question 1- Nonrandomized studies* | | | | |  |  |
| Kubba 1966^3^ |  |  |  |  |  |  |
| Prema 1982^4^ |  |  |  |  | Key: |  |
| Delgado Betancourt 1984^5^ |  |  |  |  |  | Low risk of bias |
| Diaz 1985^6^ |  |  |  |  |  | Moderate risk of bias |
| Wongubol 2010^7^ |  | BF  I  |  |  |  | High risk of bias |
| Dutta 2013^8^ |  |  |  |  |  |  |
| Parker 2021^9^ |  |  |  |  |  |  |

| **Study** | **Randomization** | **Deviations** | **Missing Data** | **Measurement** | **Reporting** | **Overall risk of bias** |
| --- | --- | --- | --- | --- | --- | --- |
| *Question 2- Randomized controlled trials* | | | | | | |
| Carmo 2017^10^ |  |  |  |  |  |  |
| Averbach 2019^11^ |  |  | BF  I  |  |  | BF  I  |

Abbreviations: BF = breastfeeding outcomes, I = infant outcomes

References:

1. Sinchai W, Sethavanich S, Asavapiriyanont S, et al. Effects of a progestogen-only pill (Exluton) and an intrauterine device (Multiload Cu250) on breastfeeding. *Adv Contracept*. 1995;11(2):143-155.

2. Braga GC, Ferriolli E, Quintana SM, Ferriani RA, Pfrimer K, Vieira CS. Immediate postpartum initiation of etonogestrel-releasing implant: A randomized controlled trial on breastfeeding impact. *Contraception*. 2015;92(6):536-542. doi:10.1016/j.contraception.2015.07.009

3. Kubba K. The effect of oral progestagens on lactation. *J*. 1966;Fac. Med. 8(2):66-69.

4. Prema K. Duration of lactation and return of menstruation in lactating women using hormonal contraception and IUDs. *Contracept Deliv Syst*. 1982;3(1):39-46.

5. Delgado Betancourt J, Sandoval JC, Sanchez F, Vallesteros De Cano P, De La Luz Bantista M, Jimenez F. Influence of Exluton (progestogen-only OC) and the Multiload Cu 250 IUD on lactation. *Contracept Deliv Syst*. 1984;5(2):91-95.

6. Diaz S, Herreros C, Juez G, Peralta O, Croxatto HB. [Influence of Norplant contraceptive implants on lactation and infant growth]. *Rev Chil Obstet Ginecol*. 1985;50(5):421-428.

7. Wongubol P. The Different Effect of a Progestogen-only Pill and Intrauterine Device Contraception on Breast Milk Volume and Infant Growth. *Reg 4-5 Med J วารสารแพทย์เขต 4-5*. 2010;29(3):303‐314.

8. Dutta DK, Dutta I. Desogestrel mini pill: is this safe in lactating mother? *J Indian Med Assoc*. 2013;111(8):553-555.

9. Parker LA, Sullivan S, Cacho N, Krueger C, Mueller M. Effect of Postpartum Depo Medroxyprogesterone Acetate on Lactation in Mothers of Very Low-Birth-Weight Infants. *Breastfeed Med Off J Acad Breastfeed Med*. 2021;16(10):835-842. doi:10.1089/bfm.2020.0336

10. Carmo L, Braga GC, Ferriani RA, Quintana SM, Vieira CS. Timing of Etonogestrel-Releasing Implants and Growth of Breastfed Infants: A Randomized Controlled Trial. *Obstet Gynecol*. 2017;130(1):100-107. doi:10.1097/AOG.0000000000002092

11. Averbach S, Kakaire O, McDiehl R, Dehlendorf C, Lester F, Steinauer J. The effect of immediate postpartum levonorgestrel contraceptive implant use on breastfeeding and infant growth: a randomized controlled trial. *Contraception*. 2019;99(2):87-93. doi:10.1016/j.contraception.2018.10.008
